# Effects of gravel on the water infiltration process and hydraulic parameters of stony soil in the eastern foothills of Helan Mountain, China

**DOI:** 10.1038/s41598-024-60364-4

**Published:** 2024-07-16

**Authors:** Yan Ma, Youqi Wang, Yuhan Zhang, Ruiyuan Zhang, Cheng Yuan, Chengfeng Ma, Yiru Bai

**Affiliations:** 1 Breeding Base for State Key Lab of Land Degradation and Ecological Restoration in Northwestern China, Ningxia University, Yinchuan, 750021 China; 2 School of Geography and Planning, Ningxia University, Yinchuan, 750021 China; 3 School of Ecology and Environment, Ningxia University, Yinchuan, 750021 China

**Keywords:** Gravel content, Hydraulic parameters, Hydrus-1D, Stony soil, Water infiltration process, Ecology, Hydrology

## Abstract

The investigation into the impact of gravel on water infiltration process and hydraulic parameters in stony soil could offer a theoretical basis to enhance water availability in rocky mountain area. A one-dimensional vertical infiltration experiment was used in this study. Six groups of gravel content of 0% (CK), 10% (W1), 20% (W2), 30% (W3), 40% (W4) and 50% (W5) were established to explore the changes in the wetting front, cumulative infiltration volume and infiltration rate. Then the accuracy of four infiltration models in simulating soil water infiltration processes was evaluated. Finally, Hydrus-1D was used to perform numerical inversion of the soil water content after infiltration. The findings revealed that: (1) When the infiltration time reached 300 min, the wetting front of the W1_,_ W2_,_ W3, W4 and W5 treatments was 11.00%, 17.00%, 32.25%, 38.75% and 54.50% lower than CK, the cumulative infiltration volume was 29.80%, 38.97%, 45.62%, 54.74% and 73.17% lower than CK, and the stable infiltration rate was 50.98%, 52.94%, 66.67%, 68.63% and 86.27% lower than CK. (2) The soil–water infiltration processes were accurately described by the Horton model, the coefficient of determination (*R*^2^) > 0.935. (3) The simulation results of Hydrus-1D showed that with the increase of gravel content, the values of the retention water content (*θ*_*r*_), saturated water content (*θ*_*s*_), shape coefficient (*n*) and saturated hydraulic conductivity (*K*_*s*_) were decreased, the values of the reciprocal of air-entry (*α*) were increased. The value of *R*^2^ was more than 0.894, the root mean square error (*RMSE*) and mean absolute error (*MAE*) were less than 2%, which demonstrated that the Hydrus-1D model exhibited superior capability in simulating the changes of water content in stony soil in rocky mountain area. The findings of this study demonstrated that gravel could decrease the water infiltration process and affect the water availability. It could provide data support for the water movement process of stony soil and rational utilization of limited water resources in mountainous area.

## Introduction

Helan Mountain is an important natural geographic demarcation line in China and an important ecological security barrier in Northwest China, guarding the ecological security of Northwest and North China^1^. At the same time, Helan Mountain is located in the arid region of Northwest China with dry climate, low rainfall, high evaporation and low soil water content^2^. Drought and water scarcity are the key factors contributing to poor crop quality and low water utilization in the region^3,4^. Therefore, effective utilization of water resources was one of the important ways to promote ecological restoration and agricultural development in the eastern foothills of the Helan Mountain^4^. As an important component of water circulation, the soil water infiltration could affect the soil runoff and erosion, and was important for water redistribution and improving water use efficiency^5^.

The process of soil water infiltration is affected by a combination of various factors, including the type of soil texture^6^, soil structure^7^, soil water content^8,9^, soil physicochemical properties^10,11^, land use^12,13^, and vegetation type^14^. Gravel in soil could have an effect on soil properties and structure, and then affect soil infiltration processes^15^. Moreover, the water-crossing profile and water movement channels would be affected by gravel^16–18^, thus the water infiltration process in stony soil was more complex than homogeneous soil^19^. The long-term water and wind erosion was accompanied by human mining activities in Helan Mountain, resulting in gravel embedded in the soil or overlying the surface^20,21^. Therefore, it was necessary to clarify the water infiltration process and estimate the hydraulic parameters of stony soil^22^, which had great significance for the efficient use of soil and water resources and the promotion of ecological restoration in the eastern foothills of the Helan Mountain^23,24^.

In recent years, many scholars have conducted some researches on the impact of gravel on soil water infiltration process and water holding properties in different regions. For example, in south-central Poland, Ilek et al.^25^ revealed that the increasing or decreasing of soil water infiltration capacity depended on the gravel content and diameter. In the central cordillera of Colombia, Leal et al.^26^ showed that infiltration was associated with the changes in total surface porosity and gravel. In the Inner Mongolia, Wu et al.^27^ found that with the increase of the gravel content and diameter, the infiltration capacity of soil water would be reduced, the water retention capacity of stony soil would be enhanced, and the soil water infiltration process was accurately described by the Kostiakov model. In Shaanxi Province, Li et al.^28^ revealed that there was a negative correlation between the gravel content and both the mean runoff rate and flow rate. In the Loess Plateau of China, Dong et al.^29^ found that with the increase of the sandstone, the water infiltration process was decreased, while the soil capacity to retain water was increased, and the Philip model fitting result was the optimal choice. All the above studies demonstrated that the infiltration process and water retention properties of stony soil were affected by the gravel content and diameter. However, the effect of gravel content on the water infiltration process and water retention capacity of stony soil was not yet understood in the eastern foothills of Helan Mountain.

Hydrus-1D was a numerical model developed by the U.S. Salinity Laboratory^30^, the inversion parameters of which were numerically simulated by one-dimensional unsaturated soil water motion equation^31^ and fixed solution conditions^32^. Ivonir et al.^33^ used Hydrus-1D combined with the water content (*θ*) and pressure head (*h*) to invert and optimize the hydraulic parameters, the simulation result of which was better. Tadesse et al.^34^ used Hydrus-1D to simulate soil water content and soil temperature combining data on precipitation, evaporation, and average temperature. Ma et al.^35^ used infiltration data and Hydrus-1D model to simulate and analyze the impact of humic acid addition on hydraulic parameters, the results of which showed that simulation results met the accuracy requirements. However, there were few studies using infiltration data and Hydrus-1D model to invert the hydraulic parameters of stony soil in the eastern foothills of Helan Mountain.

The primary aims of this research were as follows (1) to explore the changes in the stony soil water infiltration process under different gravel content; (2) to evaluate the applicability of four infiltration models in study area; (3) to simulate the soil profile water content by Hydrus-1D. These results could offer a theoretical basis for grasping the water movement status of stony soil and rational utilization of limited water resources in mountainous area.

## Materials and methods

### Materials

**Figure 1:**
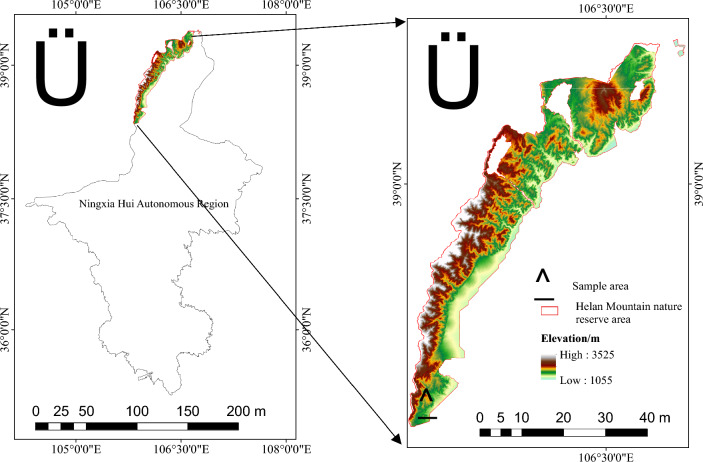
The location of the sample area. Note:Maps were created in ArcGIS 10.8, the Ningxia Hui Autonomous Region map (in .json format) was sourced from aliyun data visualisation platform https://datav.aliyun.com/portal/school/atlas/area_selector and the format conversion to shapefile was at mapshaper https://mapshaper.org/. DEM was sourced from Geospatial Data Cloud https://www.gscloud.cn/search.

The test soil sample and gravel were taken in April 2023 from the eastern foothills of Helan Mountain, Ningxia Hui Autonomous Region (105° 58′ E, 38° 23′ N, Fig. 1), and the climate was temperate continental. The mean annual temperature was 10.1 °C, the precipitation was 179.3 mm and the evaporation was 1946.1 mm. And the mean annual frost-free period and sunshine time was 178 d and 3044.1 h, respectively. The main peak had an elevation of 3,556 m, and the slope was from 0 to 72.37°. The soil physical and chemical properties were showed in Table 1. According to the classification of WRB for Soil Resources, the soil type of this paper was a hyperochric. The soil samples were collected from the top soil layer (0–40 cm) using tools such as soil augers and shovels, and the number of samples was five. The soil sample was separated through a 2 mm sieve and removed impurities. The gravel was smooth oval hard gravel, almost impermeable to water, and passed through a stainless-steel sieve with a diameter of 2.5–3.0 cm. The gravel was washed and air-dried and prepared for use.

**Table 1:** Soil physical and chemical properties.

	Clay (%)	Silt (%)	Sand (%)	pH	SOM (g kg^−1^)	TN (g kg^−1^)	TP (g kg^−1^)	EC (μs cm^−1^)	BD (g cm^−3^)
Hyperochric soil	4.20 ± 0.45	54.0 ± 1.65	41.4 ± 1.52	8.23 ± 0.04	1.62 ± 0.06	0.42 ± 0.02	0.33 ± 0.02	635 ± 87.23	1.50 ± 0.02

Data in the table were means ± standard deviation (n = 3); *SOM* Soil organic matter, *TN* Total nitrogen, *TP* Total phosphorus, *EC* Electrically conductivity, *BD* Soil bulk density.

### Methods

**Figure 2:**
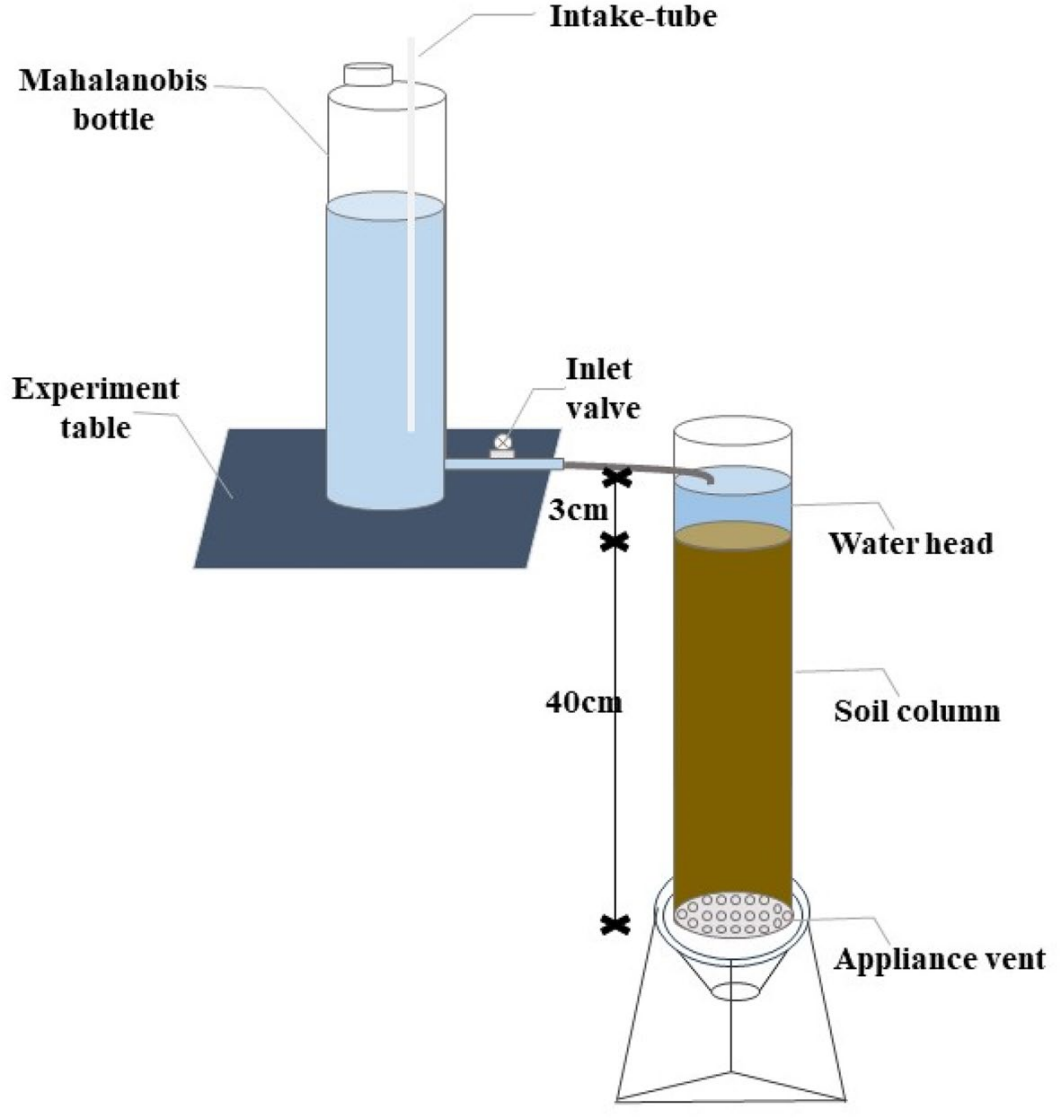
Equipment for one-dimensional vertical infiltration experiment.

The infiltration experiment was conducted using the one-dimensional vertical constant head method (Fig. 2). The different content of gravel was evenly mixed with the soil and filled in the Plexiglas columns, which had a diameter of 10 cm, a height of 50 cm, a wall thickness of 0.5 cm and a bottom thickness of 1 cm. The exhaust holes (with a diameter of 2 mm) were uniformly distributed at the bottom for easy ventilation. A water-supply Mahalanobis bottle (with a diameter of 10 cm and a height of 50 cm) was used to supply water, and the water head was controlled at about 3 cm when the water was supplied vertically.

To study the infiltration process in the stony soil with different gravel content, gravel was selected with a diameter range of 2.5–3.0 cm and mixed with the tested soil at the content of 0, 10%, 20%, 30%, 40% and 50%. The experiment was conducted in triplicate for each treatment, a total of 18 treatments were established. Soil and gravel samples were weighed and mixed well in layers (5 cm) with soil sample bulk density of 1.5 g cm^−3^ and gravel bulk density of 2.87 g cm^−3^, and then packed in Plexiglas columns. In order to avoid soil stratification, the soil was fluffed between layers, a layer of filter paper was used to tile on the bottom of the soil column before filling, a layer of filter paper was used to cover the soil surface after filling the soil column. Water was supplied in a Mahalanobis bottle with a head control of about 3 cm.

During the experiment, the value of water surface height in the Mahalanobis bottle and the change in vertical depth of the wetting front were observed and recorded at various time intervals. The values were the averages of 4 scale readings taken from the anterior, posterior, left and right aspects of the Mahalanobis bottle. For the first 5 min, the data were recorded every 10 s. For the period 5–10 min, the observations were made every 30 s. For 10–20 min, the data were recorded every minute. For 20–30 min, the observations were made every 5 min. For the period 30–60 min, the data were recorded 10 min, and after 60 min, the data were recorded every 30 min. As soon as the wetting front was transported to the bottom of the soil column, the Mahalanobis bottle water supply was immediately stopped, and the water was quickly drained, the final date of the Mahalanobis bottle was recorded.

### Infiltration model

In this paper, four commonly models were used to simulate the infiltration rate of different soil gravel content, the Philip model^36–38^, the Horton model^39,40^, the Kostiakov model^41,42^ and the General Empirical model, and compare the suitability of four infiltration models.

1. The Philip model equation
  1 $$i = 0.5St^{ - 0.5} + i_{c}$$
  where *i* is the infiltration rate, cm min^−1^; *S* is the soil infiltration rate, cm min^−0.5^; *t* is the time, min; *i*_*c*_ is the stable infiltration rate, cm min^−1^.
2. The Horton model equation
  2 $$i = i_{c} + (i_{1} - i_{c} )e^{ - kt}$$
  where *i*_1_ is the initial infiltration rate, cm min^−1^; *k* is the parameter of the infiltration model; Other parameters are as stated previously.
3. The Kostiakov model equation
  3 $$i = ct^{ - b}$$
  where *c* and *b* are model parameters; *c* represents the initial infiltration rate, cm min^−1^; *b* represents the degree of decline in water infiltration rate.
4. The General Empirical model equation
  4 $$i = v + ft^{ - w}$$
  where *v*, *f* and *w* are model parameters, the absolute value of *v* represents the stable infiltration rate, cm min^−1^, the absolute value of *f* represents the initial infiltration rate, cm min^−1^.

### Hydrus-1D basic equation

At the end of the soil infiltration process, the soil was taken in layers from top to bottom every 5 cm sequentially and the soil volumetric water content was determined. In Hydrus-1D, the soil profile water content was simulated mainly determined by means of a one-dimensional unsaturated soil water motion equation (Richards)^31^ and fixed solution conditions^32^. The equation is as follows:

5 $$C\left( h \right)\frac{\partial h}{{\partial t}} = \frac{\partial }{\partial z}\left[ {k(h)\left( {\frac{\partial h}{{\partial z}} - 1} \right)} \right]$$

The van Genuchten–Mualem model was required to simulate the soil moisture characteristic curve *θ*(*h*) and the soil non-saturated water conduction *K*(*h*) The model is expressed as:

6 $$\theta (h) = \left\{ {\begin{array}{*{20}c} {\theta_{r} + \frac{{\theta_{s} - \theta_{r} }}{{\left[ {1 + \left| {\alpha h} \right|^{n} } \right]^{m} }}} & {h < 0} \\ {\theta_{s} } & {h \ge 0} \\ \end{array} } \right.$$

7 $$K(h) = KsS_{e}^{l} \left[ {1 - (1 - S_{e}^{\frac{1}{m}} )^{m} } \right]^{2}$$

8 $$S_{e} = \frac{{\theta - \theta_{r} }}{{\theta_{s} - \theta_{r} }}$$

where *θ*_*r*_ is the retention water content, cm^3^ cm^−3^; *θ*_*s*_ is the saturated water content, cm^3^ cm^−3^; *K(h)* is the non-saturated hydraulic conductivity, cm min^−1^; *K*_*s*_ is the saturated hydraulic conductivity, cm min^−1^; *α* is a parameter related to the suction power, 1 cm^−1^; m and n are the shape coefficient; *C(h)* is the ratio of water, 1 cm^−1^; *S*_*e*_ is the saturation; *t* is the time, min; *z* is the soil depth, cm; and *h* is the pressure water head, cm; Other parameters are as previously stated.

During the infiltration test, the simulated depth of the soil layer was 40 cm, the water head was set to 3 cm, and the observation points were arranged at 2.4, 7.6, 12.4, 17.6, 22.4, 27.6, 32.4, and 37.6 cm, respectively. The pressure head was set as the upper boundary of the model and the free drainage as the lower boundary of the model.

The initial conditions were:

9 $$\begin{array}{*{20}c} {h = h_{0} (z)} & {0 \le z \le L} & {t = 0} \\ \end{array}$$

The boundary conditions were:

10 $$\left\{ {\begin{array}{*{20}l} {h = h_{1} } \hfill & {z = 0} \hfill & {t \ge 0} \hfill \\ {\frac{\partial h}{{\partial z}} = 0} \hfill & {z = L} \hfill & {t > 0} \hfill \\ \end{array} } \right.$$

where *h*_0_*(z)* is the initial pressure head, cm, *h*_1_ is defined upper pressure head and *z* is the depth of water on the surface of the soil (3 cm), *L* is the height of the soil column, cm.

### Model evaluation

The results of the forward fitting of the parameters obtained from the inversion were evaluated using three error analysis indexes: the coefficient of determination (*R*^2^), the root mean square error (*RMSE*), and the mean absolute error (*MAE*)^43^. The specific calculation formula as follows:

11 $$R^{2} = 1 - \frac{{\sum\limits_{i = 1}^{m} {\left( {M_{i} - E_{i} } \right)^{2} } }}{{\sum\limits_{i = 1}^{m} {\left( {M_{i} - \overline{{M_{i} }} } \right)}^{2} }}$$

12 $$RMSE = \sqrt {\frac{1}{m}\sum\limits_{i = 1}^{m} {(E_{i} - M_{i} )^{2} } }$$

13 $$MAE = \frac{1}{m}\sum\limits_{i = 1}^{m} {\left| {E_{i} - M_{i} } \right|}$$

where *E*_*i*_ represents the simulated value, *M*_*i*_ represents the measured value, ${\overline{M}}_{i}$ represents the average of the measured values, and *m* represents the number of samples.

### Data processing and analysis

Excel 2010 and Origin 2022 were used for graphing and data fitting; IBM SPSS Statistics 27 software was used for data analysis. The differences in the infiltration process among different treatments were analyzed using an analysis of variance (ANOVA) and Least Significant Difference (LSD) method. The number of replicates were *n* = 3, and the significance level was *p* < 0.05. *R*^2^, *RMSE* and *MAE* were used to evaluate the inversion results of soil hydraulic parameters.

## Results and analysis

### Effect of gravel content on the wetting front

**Figure 3:**
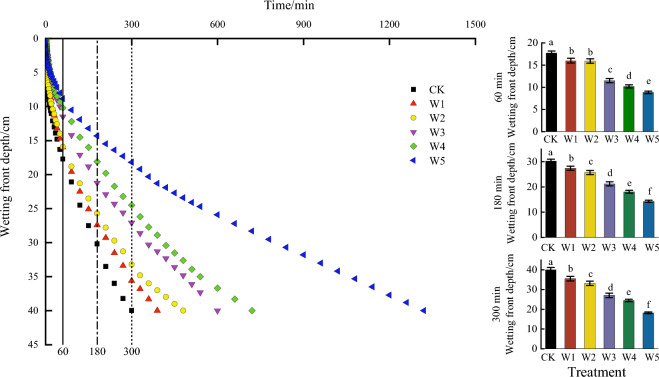
Effect of gravel content on wetting front. *Note*: Different lowercase letters indicated that the statistical significance of the wetting front was observed among different experimental treatments at the 0.05 level.

The whole infiltration process was divided into three stages based on the change of the infiltration rate^17^, which was initial infiltration stage (0–60 min), infiltration stage (60–180 min) and stable infiltration stage (180–300 min). The wetting front migration with different gravel content was showed in Fig. 3. It could be seen that the migration rate of wetting front under different treatments showed a tendency to be faster and then slower as the infiltration time increasing. The greater of the gravel content, the slower the process of wetting front migration, and the difference was more obvious.

At the beginning of the infiltration, the difference among different treatments at 60 min was significant except W1 and W2 (*p* < 0.05). As the infiltration time progressed, the influence of different gravel content on the infiltration process gradually became obvious. When the infiltration time reached 180 min, the wetting front of the W1_,_ W2_,_ W3, W4 and W5 treatments was 9.270%, 14.90%, 29.80%, 40.07% and 52.65% significantly lower than CK (*p* < 0.05). When the infiltration time reached 300 min, the wetting front of the W1_,_ W2_,_ W3, W4 and W5 treatments was 11.00%, 17.00%, 32.25%, 38.75% and 54.50% significantly lower than CK (*p* < 0.05).

The greater of the gravel content, the slower of the wetting front migration rate, indicating that the increase of the gravel content had an increased hindrance to water infiltration. The time of the wetting front reaching 40 cm depth for CK, W1_,_ W2_,_ W3, W4 and W5 was 300, 390, 480, 600, 720, and 1,320 min, respectively. This result indicated that the time taken for the wetting front to the maximum distance increased with the increase of the gravel content.

### Effect of gravel content on the cumulative infiltration volume

**Figure 4:**
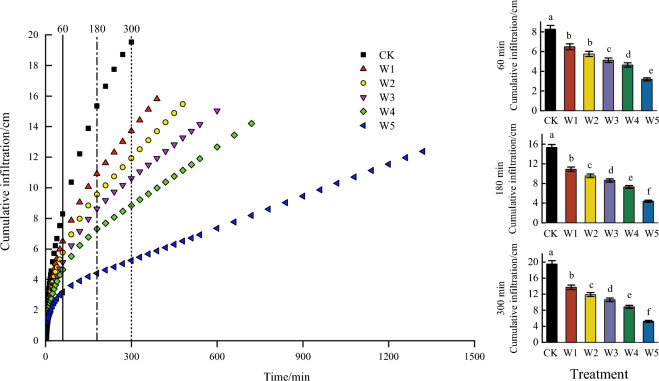
Effect of gravel content on cumulative infiltration volume. *Note* Different lowercase letters indicated that the statistical significance of the cumulative infiltration volume was observed among different experimental treatments at the 0.05 level.

The time stage of the cumulative infiltration volume was consistent with the wetting front. Figure 4 showed the change in the cumulative infiltration volume with different gravel content.

As the infiltration process proceeded, there was a significant difference among different treatments at 60 min except W1 and W2 (*p* < 0.05). When the infiltration time reached 180 min, the cumulative infiltration volume of the W1_,_ W2_,_ W3, W4 and W5 treatments was 28.83%, 37.66%, 43.71%, 52.37% and 71.30% significantly lower than CK (*p* < 0.05). When the infiltration time reached 300 min, the cumulative infiltration volume of the W1_,_ W2_,_ W3, W4 and W5 treatments was 29.80%, 38.97%, 45.62%, 54.74% and 73.17% significantly lower than CK (*p* < 0.05).

At the end of the infiltration, the cumulative infiltration volume was 19.53, 15.81, 15.47, 15.04, 14.20 and 12.38 cm on different treatments, respectively. The results showed a trend of cumulative infiltration decreasing with the increase of gravel content.

### Effect of gravel content on the infiltration rate

**Figure 5:**
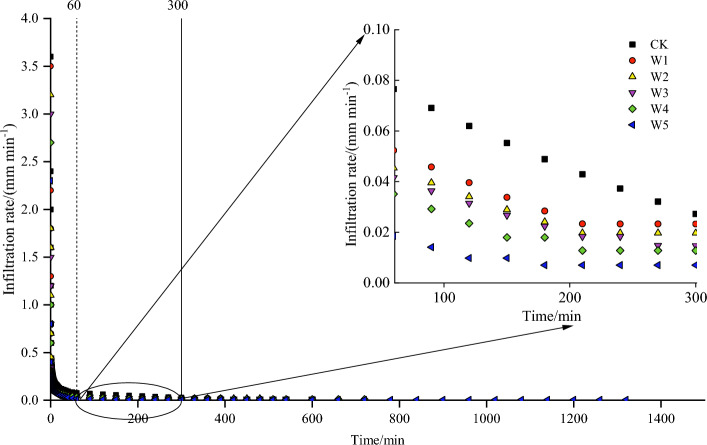
Effect of gravel content on infiltration rate.

The time stage of the infiltration rate was also consistent with the wetting front. The soil infiltration rate was a direct indicator characterizing the infiltration capacity of soil water. Figure 5 and Table 2 showed the impact of different gravel content on the change of infiltration rate. As could be seen in Fig. 5, the infiltration rate all exhibited a decreasing trend with the increase of gravel content, and the greater the gravel content, the slower the infiltration rate. However, the degree of reduction in infiltration rate varied for different infiltration time. During the initial infiltration phase, the infiltration rate of all treatments was greater and the rate of decline was faster, and the differences of all treatments were not obvious. With the infiltration process progressed, the rate of decline slowed down and the differences among all treatments gradually became obvious.

**Table 2:** Effect of different gravel content on water infiltration rate in stony soil.

Treatment	${i}_{1}$/(mm min^−1^)	${i}_{c}$/(mm min^−1^)	$\overline{i}$/(mm min^−1^)
CK	0.389 ± 0.1220a	0.051 ± 0.0185a	0.255 ± 0.0027a
W1	0.341 ± 0.1149ab	0.025 ± 0.0030b	0.204 ± 0.0004b
W2	0.309 ± 0.1054ab	0.024 ± 0.0040b	0.174 ± 0.0005c
W3	0.266 ± 0.0888ab	0.017 ± 0.0020bc	0.143 ± 0.0005d
W4	0.237 ± 0.0725ab	0.016 ± 0.0040bc	0.123 ± 0.0004e
W5	0.189 ± 0.0645b	0.007 ± 0.0005c	0.081 ± 0.0004f.

Different lowercase letters indicated that the statistical significance of the infiltration rate was observed among different experimental treatments at the 0.05 level.

In Table 2, the infiltration rate during the initial infiltration phase was higher, and the significant differences were found between CK and W5 (*P* < 0.05). The *i*_1_ of the W1_,_ W2_,_ W3, W4 and W5 treatments was 12.34%, 20.57%, 31.62%, 39.07% and 51.41% lower than CK. As the infiltration process proceeded, the infiltration rate was constantly decreasing. At 300 min, the infiltration process reached the stable infiltration stage, the significant differences of infiltration rate were found between CK and other treatments (*P* < 0.05). The *i*_c_ of the W1, W2, W3, W4 and W5 treatments was 50.98%, 52.94%, 66.67%, 68.63% and 86.27% lower than CK. For the $\overline{i}$, significant differences were found among all treatments (*P* < 0.05).

### Fitting model of soil water infiltration process

In order to investigate the effect of different gravel content on the soil water infiltration process, the Philip model, Horton model, Kostiakov model and General Empirical model were selected to simulate the water infiltration process.

As shown in Table 3, the *R*^2^ in the Philip model was between 0.667 and 0.712, *S* was between 0.276 and 0.469. The *S* represented different water infiltration capacities, and larger values indicated a greater infiltration capacity. The values of *S* decreased sequentially from CK to W5, which indicated that the gravel had an inhibitory impact on the water infiltration process, the higher the gravel content, the greater the inhibition effect on water infiltration processes. The *R*^2^ values in Horton model were all greater than 0.935, the values of *i*_*c*_ and *i*_1_ decreased with the increase of the gravel content, consistent with the pattern of change in the measured data. The *R*^2^ values in Kostiakov model were between 0.872 and 0.888. From the parameters *c* and *b*, it could be seen that an increase in gravel content would result in the decreasing of the initial infiltration rate, accelerate the recession degree of infiltration rate and slow down the water infiltration process. The *R*^2^ values in General Empirical model were all greater than 0.941. The absolute value of the parameters *v* and *f* represented the stable infiltration rate and initial infiltration rate of soil water, respectively. It could be seen that *v* and *f* both showed a decreasing trend with the increase of the gravel content. In a comprehensive comparison, the descending order of the *R*^2^ values was Horton model > General Empirical model > Kostiakov model > Philip model.

**Table 3:** Parameters of soil water infiltration model fitting under different gravel content.

Model	Parameters	CK	W1	W2	W3	W4	W5
Philip model	*S/*(cm min^−0.5^)	0.469	0.449	0.421	0.367	0.321	0.276
	*i*_*c*_/(cm min^−1^)	0.128	0.091	0.074	0.060	0.053	0.047
	*R*^2^	0.684	0.700	0.711	0.712	0.695	0.667
Horton model	*i*_*c*_/(cm min^−1^)	0.078	0.051	0.041	0.033	0.026	0.020
	*i*_1_/(cm min^−1^)	0.578	0.519	0.471	0.400	0.340	0.259
	*k*	0.175	0.178	0.176	0.165	0.141	0.080
	*R*^2^	0.981	0.983	0.983	0.981	0.981	0.935
Kostiakov model	*c*/(cm min^−1^)	0.412	0.360	0.325	0.280	0.247	0.217
	*b*	0.272	0.293	0.295	0.297	0.298	0.299
	*R*^2^	0.875	0.872	0.877	0.882	0.877	0.888
General empirical model	*v*/(cm min^−1^)	− 0.686	− 0.398	− 0.282	− 0.223	− 0.217	− 0.207
	*f*/(cm min^−1^)	1.125	0.783	0.629	0.521	0.480	0.438
	*w*	0.090	0.122	0.140	0.144	0.132	0.093
	*R*^2^	0.947	0.942	0.941	0.945	0.946	0.969

### Inversion of soil hydraulic parameters and simulation of water content distribution based on Hydrus-1D

**Figure 6:**
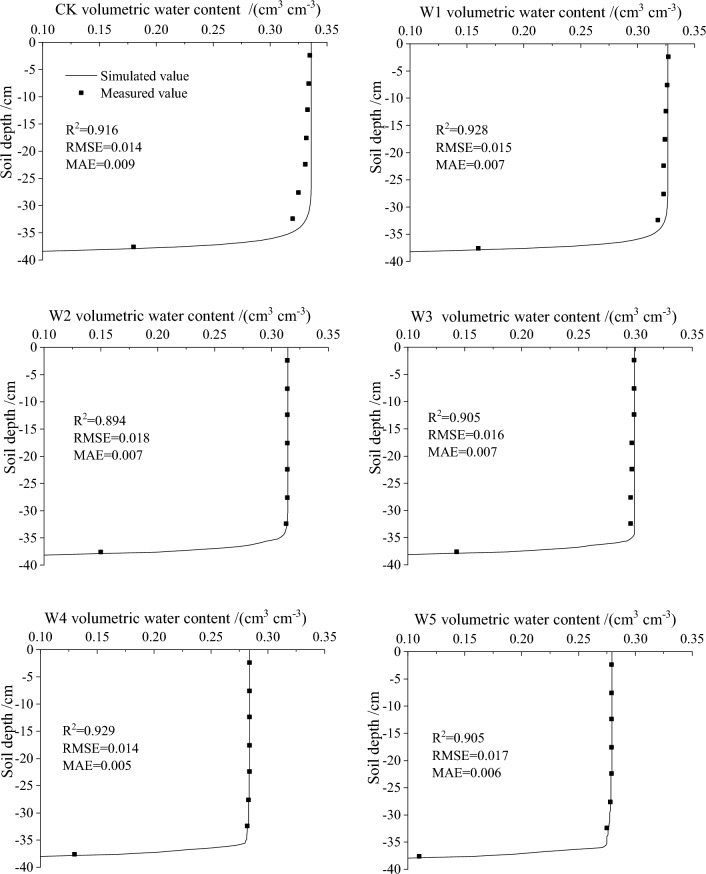
Comparison of simulated and measured values of soil water content distribution for different gravel content.

As illustrated in Fig. 6, the soil water content of all treatments decreased progressively with the increase of the soil depth. And the water content at the same layer of soil profile decreased with the increase of gravel content in different treatments. At the end of the infiltration, the soil volumetric water content of W1_,_ W2_,_ W3, W4 and W5 at 5–10 cm was 0.334, 0.314, 0.299, 0.284 and 0.279 cm^3^ cm^−3^, respectively, and 2.40%, 5.99%, 10.48%, 14.97% and 16.47% lower than CK.

In this paper, the cumulative infiltration data obtained from the one-dimensional vertical infiltration experiment were combined with the prescribed initial and boundary conditions. The inversion module in Hydrus-1D was used to the inversion of the hydraulic parameters (*θ*_*r*_, *θ*_*s*_, *α*, *n* and *K*_*s*_) in the van Genuchten–Mualem model. The inversion results of soil hydraulic parameters were showed in Table 4, which showed that *θ*_*r*_, *θ*_*s*_*, n* and *K*_*s*_ decreased, while *α* increased with the increase of the gravel content.

**Table 4:** Results of inversion of soil hydraulic parameters with different gravel content.

Treatment	${\theta }_{r}$/(cm^3^ cm^−3^)	${\theta }_{s}$/(cm^3^ cm^−3^)	*α/*(1 m^−1^)	*n*	${K}_{s}$/(cm min^−1^)	*l*
CK	0.028	0.330	0.101	2.500	0.018	0.500
W1	0.024	0.319	0.125	2.460	0.015	0.500
W2	0.021	0.308	0.151	2.420	0.013	0.500
W3	0.018	0.297	0.330	2.380	0.012	0.500
W4	0.011	0.283	1.621	2.250	0.011	0.500
W5	0.008	0.279	1.634	2.130	0.006	0.500

The simulation results of the soil volumetric water content were showed in Fig. 6.The *R*^2^, *RMSE*, and *MAE* were used to assess the fitting degree between the simulated value and the measured value. The values of *R*^2^ were between 0.894 and 0.929, the values of *RMSE* and *MAE* were both less than 2%, which showed that the difference between the simulated and measured values was very small and the fitting effect was better.

## Discussion

### Effects of gravel content on the wetting front, the cumulative infiltration volume and infiltration rate

The water infiltration in stony soil with different gravel content was investigated in this paper, which found that during the initial phase of infiltration process, the wetting front, cumulative infiltration volume, and infiltration rate showed a rapid increase in all treatments. As the infiltration process progressed, trends in the curves were flattening. The wetting front, cumulative infiltration volume and infiltration rate showed the similar change rules with the increase of the gravel content. However, the infiltration time required to reach the same depth increased with the increase of the gravel content, which showed the greater gravel content, the stronger the hindering effects on the soil water infiltration process, in this paper the most effective gravel content for reducing soil permeability was 50%. This was consistent with the results of Wu et al.^27^, who studied the water infiltration process of stony soil in Inner Mongolia. The gravel in the soil favored the reduction of the contact area between soil fine particles, which would lead to an accumulation of fine particles around it^26^. Then the total soil porosity would reduce and the soil bulk density would increase, both of which were important influence factors in determining soil water infiltration. Wang et al.^44^ studied the soil infiltration and found the similar conclusion, which showed that the higher soil bulk density and the lower total soil porosity and capacity.

Meanwhile, the decrease in the wetting front was due to the total soil porosity decreased with the increase of the gravel content, as a result the water flow pathways conditions were poor and the permeability of the soil was reduced. Ilek et al.^25^ stated that the gravel increased the resistance in the soil compared to fine grained soils (< 2 mm). The decrease in the cumulative infiltration volume was due to the fact that the soil structure also became more compact with the increase of the gravel content, and the soil bulk weight became larger^45–47^, the water infiltration was prevented and the hydraulic conductivity decreased^48^. The study of^29^ on soil water infiltration in sandstone overlying loess in the Loess Plateau region also reached the same conclusion. At the initial infiltration stage, the infiltration rate was fast. This was because the presence of gravel, which could lead to the creation of preferential flow, so the water would penetrate rapidly along the pores. As the infiltration process proceeded, the infiltration rate decreased with the increase of gravel content, it could be seen in Fig. 4. The reason for this phenomenon could be that in the early stages of infiltration, the gravel covered on the top soil layer could protect the underlying soil from the direct influence of the water, which could promote the creation of soil aggregates^26^. While with the infiltration process, the soil aggregates were disrupted by gravel and the soil pore structure was blocked. Finally, the infiltration process was hindered and the infiltration rate were reduced^27^.

### Effect of gravel content on the fitting of four infiltration models

In this paper, four infiltration models were selected for evaluation and analysis, among which the Horton model had the best model fitting effect (*R*^2^ > 0.935). The Horton model was applicable to describe the vertical one-dimensional infiltration in unsaturated soil^49^ and the parameters *i*_*c*_ and *i*_1_ had physical significance^50–52^. And the Philip model with the lowest *R*^2^ values had the lowest accuracy among the four different infiltration models in the paper. The Kostiakov model was a typical exponential model^49^ and the simulated infiltration rate *i* tended to be infinite when the time tended to 0. It was not consistent with the actual situation^53^ and was not suitable for characterizing the water movement in the early stage of soil water infiltration processes. And the Kostiakov model was a semi-empirical model, its parameters didn’t have clear physical significance, so many scholars didn’t choose it to simulate infiltration rate^54^. At the same time, the *R*^2^ values of Kostiakov model were smaller than the Horton model and the General Empirical model, so the simulation of the Kostiakov model was less effective in comparison. Besides, General Empirical model was a mathematical model and did not reflect the soil infiltration capacity, the *R*^2^ values of which were also smaller than that of the Horton model in this study.

In summary, four infiltration models were evaluated and analyzed, in which the Horton model could better simulate the effect of gravel on the water infiltration process of stony soil in the east foothills of Helan Mountain. Within the area of Shandong Province, Sun et al.^30^ found that the Philip model could better describe the water movement process. In Inner Mongolia, Wu et al.^27^ found that the Kostiakov model could better describe the effect of gravel fragments on soil water infiltration. At Hodzo, Atta-Darkwa et al^55^ also found that the Horton model predicted soil infiltration better than the Kostiakov and Philip model under the tillage operation. The most appropriate model should be selected based on local soil status from different regions.

### Effect of gravel content on soil hydraulic parameters

The simulation of the Hydrus-1D inversion parameters and evaluation metrics revealed the negligible discrepancies between the simulated values and measured values. The differences were caused by the assumption conditions of the model and experimental error. The Hydrus-1D model assumed that gravel-bearing soils were uniformly distributed in the soil layer, but the reality was not a uniform medium. Moreover, the gravel in this paper were regularly shaped and nearly impermeable cobbles, but in fact there were a variety of gravel types in the soil. And there were also inevitable errors in the sampling path. Despite there was a discrepancy between the measured and the simulated value, the discrepancy in this study were able to meet the accuracy requirements. So it could be apply to the study of the water infiltration of the stony soil under different gravel content.

*θ*_*r*_, *θ*_*s*_*,* n, and *K*_*s*_ were negatively correlated with gravel content, while *α* was positively correlated with gravel content, which was consistent with the Dong^29^. The decrease of *θ*_*r*_ and *θ*_*s*_ could be due to the water flow tortuosity would increase with the increase of the gravel content^56^, so the water content of the soil up to a certain volume would decrease. In addition, as the spaces among the gravel were filled with the soil, which could reduce the total pore space that decreased the space for water storage. The decrease of *K*_*s*_ could be due to the clogging of soil pores by loose gravel particles^57^, which resulted in a decrease in total soil porosity^58–60^, slowed down the soil water infiltration process and led to a decrease in *K*_*s*_. Moreover, the gravel could lead to the lateral transport of water from vertical infiltration^29^, which would reduce the effective cross-sectional area for infiltration and the redistribution of water^61^. From the above all discussion, we could conclude that the greater the gravel content, the lower the hydraulic conductivity and water holding capacity.

## Conclusions

1. The study of the gravel effect on the water infiltration process and hydraulic parameters of stony soil in the eastern foothills of Helan Mountain was analyzed and compared. It was found that under different gravel content, the wetting front, cumulative infiltration volume, and infiltration rate decreased gradually with the increase of the gravel content: CK > W1 > W2 > W3 > W4 > W5.
2. Evaluating and analyzing the actual physical significance and the fitting effect of four infiltration models (Philip model, Horton model, Kostiakov model and Generalized Empirical model), it was found that the Horton model had a better fitting effect (*R*^2^ > 0.935), which could better describe the effect of gravel on the water infiltration process of stony soil (*R*^2^ > 0.894, *RMSE* and *MAE* < 2%).
3. Hydrus-1D was used to simulate the distribution of the soil water content after infiltration, which showed that at the same soil depth the soil profile water content decreased with the increase of gravel content. Hydrus-1D could well predict the distribution of the soil profile water content in the water infiltration process of stony soil (*R*^2^ > 0.894, *RMSE* and *MAE* < 2%).

## Data Availability

All relevant data are within the paper.
